# Spin-Orbital Conversion of a Strongly Focused Light Wave with High-Order Cylindrical–Circular Polarization

**DOI:** 10.3390/s21196424

**Published:** 2021-09-26

**Authors:** Victor V. Kotlyar, Sergey S. Stafeev, Elena S. Kozlova, Anton G. Nalimov

**Affiliations:** IPSI RAS—Branch of the FSRC “Crystallography and Photonics” RAS, Molodogvardeyskaya 151, 443001 Samara, Russia; kotlyar@ipsiras.ru (V.V.K.); sergey.stafeev@gmail.com (S.S.S.); anton@ipsiras.ru (A.G.N.)

**Keywords:** tight focusing, Richards–Wolf theory, spin Hall effect, hybrid polarization, metalens

## Abstract

We discuss interesting effects that occur when strongly focusing light with *m*^th^-order cylindrical–circular polarization. This type of hybrid polarization combines properties of the *m*^th^-order cylindrical polarization and circular polarization. Reluing on the Richards-Wolf formalism, we deduce analytical expressions that describe E- and H-vector components, intensity patterns, and projections of the Poynting vector and spin angular momentum (SAM) vector at the strong focus. The intensity of light in the strong focus is theoretically and numerically shown to have an even number of local maxima located along a closed contour centered at an on-axis point of zero intensity. We show that light generates 4*m* vortices of a transverse energy flow, with their centers located between the local intensity maxima. The transverse energy flow is also shown to change its handedness an even number of times proportional to the order of the optical vortex via a full circle around the optical axis. It is interesting that the longitudinal SAM projection changes its sign at the focus 4*m* times. The longitudinal SAM component is found to be positive, and the polarization vector is shown to rotate anticlockwise in the focal spot regions where the transverse energy flow rotates anticlockwise, and vice versa—the longitudinal SAM component is negative and the polarization vector rotates clockwise in the focal spot regions where the transverse energy flow rotates clockwise. This spatial separation at the focus of left and right circularly polarized light is a manifestation of the optical spin Hall effect. The results obtained in terms of controlling the intensity maxima allow the transverse mode analysis of laser beams in sensorial applications. For a demonstration of the proposed application, the metalens is calculated, which can be a prototype for an optical microsensor based on sharp focusing for measuring roughness.

## 1. Introduction

The rigorous description of a linearly polarized electromagnetic field at the strong focus was proposed in a classical work by Richards and Wolf [1]. Numerous follow-up publications relied on the Richards–Wolf formalism to look into the behavior of more general electromagnetic fields with various polarization states. Topics studied included the characteristics of a radially polarized electromagnetic field at the strong focus [2,3,4,5], and spin-orbital conversion at the strong focus of a circularly polarized wave [6,7,8,9]. Tightly focusing an elliptically polarized optical vortex has been studied [10,11,12,13], and a concept of cylindrical vector beams has been proposed [14], including both radially and azimuthally polarized beams. Studies of the focusing of promising beams with hybrid polarization have also been conducted [15,16,17,18,19]. For this type of polarization, the transposed Jones vector takes the form E=(exp(iδ),exp(−iδ)), where δ=αr+β, *r* is a radial variable, *α*, *β* are constant, and **E** is the initial light field. This type of hybrid polarization is linear along some radii and circular on the others, while being independent of the polar angle *φ*. A more general type of hybrid polarization was discussed in [20], where the tight focusing was analyzed for an incident field described by the polar-angle-dependent Jones vector, **E** = (cos*φ*cos*γ* − *i*cos(2*ψ* − *φ*)sin*γ*, sin*φ*cos*γ* − *i*sin(2*ψ* − *φ*)sin*γ*), where *φ* is the polar angle and *γ*, *ψ* are constant. This field was found to be either linearly or circularly polarized, depending on the specific value of the polar angle. However, no analytical relations to describe the hybrid field and projections of the Poynting vector were proposed in [20]. A field with hybrid polarization described by E=(exp(iδ)sinφ, cosφ), where *φ* is the polar angle and *δ* is constant, has also been studied [21]. It should be noted that, in this work, we discuss a more general case of polarization, of which the above-mentioned polarization is a particular case (at *m* = 1). We also note that in [21], projections of the Poynting vector were not defined analytically. Beams with arbitrary polarization represented on a Poincare unit sphere were analyzed in [22,23,24,25]. For such beams, the pre-focusing polarization vector can be represented as **E** = (exp(−*ilφ* + *iα*)cosβ + exp(*ilφ* + *iα*)sin*β*, *i*exp(−*ilφ* + *iα*)cos*β* + *i*exp(*ilφ* + *iα*)sin*β*), where *l* is the topological charge, *φ* is the polar angle, and (*α*, *β*) are (constant) angles on the Poincare unit sphere. It is worth noting that [22,23,24,25] studied these beams experimentally, offering no theoretical substantiation or expressions for the field intensity and projections of the Poynting vector at the tight focus. Tightly focusing higher order cylindrically polarized light was studied in [26,27,28,29,30], with the incident field being represented as E=(cos(pφ+α), sin(pφ+α)), where *p* is the order of cylindrical polarization and *α* is constant. Vortex beams with arbitrary topological charge *m* and *n*th-order cylindrical polarization were theoretically studied in [31]. The incident field was described by E=exp(imφ)(cos(nφ), sin(nφ)).

A distinctive feature of this work is that we are the first to analyze a new type of hybrid polarization of light that has never been studied before, in which the polarization of the incident field under analysis is represented by E=(−isin(mφ), cos(mφ)). In this case, with the changing polar angle of the initial field, polarization changes from circular, to elliptical, to linear, alternating in this manner *m* times per full circle of the polar angle. We also propose analytical relationships for the projections of the E- and H-fields at the strong focus, for the intensity distributions and the projections of the Poynting vector and spin angular momentum (SAM) vector. We experimentally demonstrate the possibility of generating a second-order hybrid beam with a vortex half-wave plate. The correspondence between the experimentally obtained beam and those studied theoretically is verified using the Jones formalism.

In this paper, we continue to study the behavior of vector and vortex light fields at the sharp focus, since this problem has not yet been exhausted, with more new optical phenomena being discovered at the sharp focus of these beams. This is due to the fact that, at the sharp focus, all six projections of the electromagnetic field make approximately the same contribution, and none can be neglected. In our previous work [32], we introduced a hybrid polarization of light that combines the properties of beams with circular and cylindrical polarization. 

In contrast to our previous study, we propose an optical scheme in the form of a Mach–Zehnder interferometer that generates a beam with hybrid polarization and an arbitrary *m*^th^ order. Using the rigorous solution of Maxwell’s equations, we also calculate a subwavelength relief of a metalens that, under illumination by linearly polarized light, generates a light field with hybrid polarization, simultaneously focusing it at a wavelength distance (532 nm). One such metalens can replace the entire optical scheme for generating and focusing a light field with hybrid polarization. At the focus of the metalens, 4*m* transverse energy flows will occur in an area with a diameter of ~1 μm. The direction of energy rotation in these energy flows will alternate (clockwise and counterclockwise). In addition, such a super-thin microlens synthesized in an amorphous silicon film with a thickness of ~154 nm and a diameter of 7 μm may serve as a prototype of a compact focus sensor for optical topography measurement of rough surfaces.

## 2. Materials and Methods

### 2.1. Intensity of Light with Hybrid Polarization at the Focus 

Let the amplitudes of the original magnetic and electric field vectors for *m*^th^-order hybrid polarization be given by:

(1) $$E=A(\theta )\left(\begin{array}{l} -i\sin m\varphi \\ \cos m\varphi \end{array}\right),\;H=A(\theta )\left(\begin{array}{l} -\cos m\varphi \\ \;-i\sin m\varphi \end{array}\right),$$

where **E** and **H** are the electric and magnetic field vectors, *m* is a positive integer defining the order of cylindrical polarization, and *A*(*θ*) is the amplitude of the original light field, which depends on the angle between the incident beam and the optical axis. The polarization of the field in Equation (1) is called hybrid because it combines the properties of *m*th-order cylindrical polarization and circular polarization. At different polar angles *φ*, the polarization of field (1) will be either circular (at φ=πn/(4m), n=1,3,5…, elliptical, or linear (at φ=πn/(2m), n=0,1,2…). From (1), it also follows that at *m* = 0, the field will be uniformly linearly polarized. Our analysis relies on the Richards–Wolf integral [1], which is given by:

(2) $$\begin{array}{l} U\left(\rho ,\psi ,z\right)=-\frac{if}{\lambda }\int_{0}^{\theta_{0}}\int_{0}^{2\pi }A\left(\theta \right)T\left(\theta \right)P\left(\theta ,\varphi \right)\times \\ \times \;\exp\left\{ik\left[\rho \sin\theta \cos\left(\varphi -\psi \right)+z\cos\theta \right]\right\}\sin\theta \;d\theta \;d\varphi , \end{array}$$

where **U**(*ρ*, *ψ*, *z*) is the electrical or magnetic field at the focal spot, *A*(*θ*) is the incident electrical or magnetic field (where *θ* is the polar angle and *φ* is the azimuthal angle), *T*(*θ*) is the apodization function (the apodization function is equal to *T*(*θ*) = cos^1/2^*θ* for an aplanatic lens and to *T*(*θ*) = cos^−3/2^*θ* for a flat diffractive lens), *k* = 2*π*/*λ* is the wavenumber, *f* is the focal length, *λ* is the incident wavelength, *α*_0_ is the maximal polar angle determined by the lens numerical aperture (NA = sin*θ*_0_), and **P**(*θ*, *φ*) is the polarization matrix for the electric and magnetic fields, respectively, given by:

(3) $$P\left(\theta ,\varphi \right)=\left[\begin{array}{c} 1+\cos^{2}\varphi \left(\cos\theta -1\right)\\ \sin\varphi \cos\varphi \left(\cos\theta -1\right)\\ -\sin\theta \cos\varphi \end{array}\right]a\left(\theta ,\varphi \right)+\left[\begin{array}{l} \sin\varphi \cos\varphi \left(\cos\theta -1\right)\\ 1+\sin^{2}\varphi \left(\cos\theta -1\right)\\ -\sin\theta \sin\varphi \end{array}\right]b\left(\theta ,\varphi \right),$$

where a(*θ*,*φ*) and b(*θ*,*φ*) are the polarization functions (1) for the *x*- and *y*-components of the incident beam, respectively. The original amplitude function *A*(*θ*) (here assumed to be real) can be either constant (for a plane incident wave) or given by a Gaussian beam:

(4) $$A(\theta )=\exp\left(\frac{-\gamma^{2}\sin^{2}\theta }{\sin^{2}\theta_{0}}\right)$$

where *γ* is constant.

Relying on the Richards–Wolf formalism [1], we can derive projections of the electric field vector at the strong focus of an aplanatic optical system from the original field (see [32] for details):

(5) $$\begin{array}{l} E_{x}=\frac{-i^{m}}{2}[I_{0,m}\sin(m\varphi )+\frac{\left(1+i\right)}{2}I_{2,m+2}\sin\left((m+2)\varphi \right)+\\ \;\;\;\;\;\;\;\;\;\;+\frac{\left(1-i\right)}{2}I_{2,m-2}\sin\left((m-2)\varphi \right)],\\ E_{y}=\frac{-i^{m}}{2}[iI_{0,m}\cos(m\varphi )-\frac{\left(1+i\right)}{2}I_{2,m+2}\cos\left((m+2)\varphi \right)+\\ \;\;\;\;\;\;\;\;\;+\frac{\left(1-i\right)}{2}I_{2,m-2}\cos\left((m-2)\varphi \right)],\\ E_{z}=\frac{i^{m}}{2}[(1+i)I_{1,m+1}\sin\left((m+1)\varphi \right)+\\ \;\;\;\;\;\;\;\;\;+(1-i)I_{1,m-1}\sin\left((m-1)\varphi \right)] \end{array}$$

and for the magnetic field vector:

(6) $$\begin{array}{l} H_{x}=\frac{i^{m}}{2}[iI_{0,m}\cos(m\varphi )+\frac{\left(1+i\right)}{2}I_{2,m+2}\cos\left((m+2)\varphi \right)-\\ \;\;\;\;\;\;\;\;\;\;-\frac{\left(1-i\right)}{2}I_{2,m-2}\cos\left((m-2)\varphi \right)],\\ H_{y}=\frac{i^{m}}{2}[-I_{0,m}\sin(m\varphi )+\frac{\left(1+i\right)}{2}I_{2,m+2}\sin\left((m+2)\varphi \right)+\\ \;\;\;\;\;\;\;\;\;+\frac{\left(1-i\right)}{2}I_{2,m-2}\sin\left((m-2)\varphi \right)],\\ H_{z}=\frac{i^{m}}{2}[(1-i)I_{1,m+1}\cos\left((m+1)\varphi \right)-\\ \;\;\;\;\;\;\;\;\;-(1+i)I_{1,m-1}\cos\left((m-1)\varphi \right)], \end{array}$$

where:

(7) $$\begin{array}{l} I_{\nu ,\mu }=\left(\frac{\pi f}{\lambda }\right)\int_{0}^{\theta_{0}}\sin^{\nu +1}(\frac{\theta }{2})\cos^{3-\nu }(\frac{\theta }{2})\cos^{1/2}(\theta )\times \\ \;\;\;\;\;\;\;\;\;\;A(\theta )e^{ikz\cos\theta }J_{\mu }(x)d\theta , \end{array}$$

where *x = kr*sin*θ*, *J_μ_*(*x*) is the Bessel function of the first kind. 

From Equation (5), the intensity distribution of the electric field in the focal plane (*z* = 0) is found as:

(8) $$\begin{array}{l} I_{m}=\frac{1}{4}\left[I_{0,m}^{2}+I_{2,m+2}^{2}+I_{2,m-2}^{2}-I_{0,m}\left(I_{2,m+2}+I_{2,m-2}\right)\right]+\\ \;\;\;\;\;\;\;\;\;+\frac{1}{2}\left(I_{1,m+1}^{2}+I_{0,m}I_{2,m+2}\right)\sin^{2}\left((m+1)\varphi \right)+\\ \;\;\;\;\;\;\;\;\;+\frac{1}{2}\left(I_{1,m-1}^{2}+I_{0,m}I_{2,m-2}\right)\sin^{2}\left((m-1)\varphi \right). \end{array}$$

At *m* = 1, Equation (8) suggests that at the focus of the first-order hybrid field (1), the intensity is given by:

(9) $$\begin{array}{l} I_{1}=\frac{1}{4}\left(I_{0,1}^{2}+I_{1,2}^{2}+I_{2,1}^{2}+I_{2,3}^{2}+I_{0,1}I_{2,1}\right)-\\ \;\;\;\;\;\;\;\;\;\;\frac{1}{4}\left(I_{1,2}^{2}+I_{0,1}I_{2,3}\right)\cos\left(4\varphi \right). \end{array}$$

From Equation (9), at the focus for the first-order hybrid field (1)—i.e., for azimuthal–circular polarization—the intensity distribution features four local maxima (at φ=±π/4,±3π/4). In the general case of an arbitrary *m*, the intensity distribution in Equation (8) has 2(*m* + 1) peaks lying on the rays formed by the polar angles φ=(π+2πn)/2(m+1), n=0,1,2,…2m+1. This follows from the fact that Equation (8) contains squared sine functions, which have 2(*m* + 1) maxima as the angle *φ* changes from 0 to 2*π*. The numerical simulation confirms the theoretical conclusions. 

### 2.2. Energy Flow at the Focus of the Light with Hybrid Polarization

We note that in the original field (1), only the longitudinal projection of the energy flow is present, because the longitudinal components of the electric and magnetic field vectors are zero, as is the transverse projection of the Poynting vector. On the other hand, the longitudinal component of the SAM vector is non-zero. Hence, due to the effect of spin-orbital conversion, a transverse energy flow may be expected to be generated at the strong focus. Below, we prove this to be the case. Let us derive projections of the Poynting vector (the energy flow):

(10) P=Re(E*×H)

where Re is the real part of the number, × denotes the vector product of two vectors, and E* denotes complex conjugation in the focal plane (*z* = 0) for the original field with hybrid polarization (Equation (1)). Substituting the projections of the electric field in Equation (5) and the magnetic field in Equation (6) into Equation (10) yields:

(11) $$\begin{array}{l} P_{x}=\frac{1}{4}[I_{0,m}\left(I_{1,m+1}+I_{1,m-1}\right)\cos\varphi +\\ \;\;\;\;\;\;\;\;\;+\;I_{1,m+1}I_{2,m-2}\cos\left((m+1)\varphi \right)\cos\left((m-2)\varphi \right)+\\ \;\;\;\;\;\;\;\;\;+\;I_{1,m-1}I_{2,m+2}\cos\left((m-1)\varphi \right)\cos\left((m+2)\varphi \right)+\\ \;\;\;\;\;\;\;\;\;+\;I_{1,m+1}I_{2,m+2}\sin\left((m+1)\varphi \right)\sin\left((m+2)\varphi \right)+\\ \;\;\;\;\;\;\;\;\;\;I_{1,m-1}I_{2,m-2}\sin\left((m-1)\varphi \right)\sin\left((m-2)\varphi \right)],\\ P_{y}=\frac{1}{4}[I_{0,m}I_{1,m+1}\sin\left((2m+1)\varphi \right)+\\ \;\;\;\;\;\;\;\;\;\;+I_{0,m}I_{1,m-1}\sin\left((2m-1)\varphi \right)+\\ \;\;\;\;\;\;\;\;\;\;+I_{1,m+1}I_{2,m-2}\cos\left((m+1)\varphi \right)\sin\left((m-2)\varphi \right)-\\ \;\;\;\;\;\;\;\;\;\;-I_{1,m-1}I_{2,m+2}\cos\left((m-1)\varphi \right)\sin\left((m+2)\varphi \right)+\\ \;\;\;\;\;\;\;\;\;\;+I_{1,m+1}I_{2,m+2}\sin\left((m+1)\varphi \right)\cos\left((m+2)\varphi \right)-\\ \;\;\;\;\;\;\;\;\;\;-I_{1,m-1}I_{2,m-2}\sin\left((m-1)\varphi \right)\cos\left((m-2)\varphi \right)],\\ P_{z}=\frac{1}{4}\left(I_{0,m}^{2}-\frac{1}{2}I_{2,m+2}^{2}-\frac{1}{2}I_{2,m-2}^{2}\right). \end{array}$$

Although the expressions for the projections of the Poynting vector in Equation (11) are quite cumbersome, they allow us to make some significant general conclusions. From Equation (11), the longitudinal energy flow is seen to be radially symmetrical at any *m*, being *φ*-independent. The on-axis energy flow will be positive and non-zero only at *m* = 0 (linear polarization): $P_{z}(r=z=0)=I_{0,0}^{2}/4$. Moreover, the on-axis projection of the Poynting vector at the focus in Equation (11) will be non-zero and negative only at *m* = −2 or *m* = 2: $P_{z}(r=z=0)=-I_{2,0}^{2}/4$. Thus, we can infer that, similar to conventional 2nd-order azimuthal polarization of the incident light [33,34], for hybrid incident polarization, a reverse energy flow also occurs at m=±2. From Equation (11) at *φ* = 0, we can derive:

(12) $$\begin{array}{l} P_{x}(\varphi =0)=\frac{1}{4}[I_{0,m}\left(I_{1,m+1}+I_{1,m-1}\right)+I_{1,m+1}I_{2,m-2}+\\ \;\;\;\;\;\;\;\;\;\;I_{1,m-1}I_{2,m+2}]>0 \end{array}$$

From Equation (11), we also find that $P_{y}(y=0)=0,\;P_{x}(\varphi =0)=-P_{x}(\varphi =\pi )$ > 0. Hence, at any *m*, on the horizontal axis *x*, the transverse energy flow is always directed along the *x*-axis in both directions from the center. It also follows from Equation (11) that on the vertical axis *y*, the transverse energy flow is directed along the *y*-axis because $P_{x}(\varphi =\pi /2)=P_{x}(\varphi =3\pi /2)=0$. Equation (11) also suggests that when passing through the zero point on the *y*-axis, the energy flow will change its sign: $P_{y}(\varphi =\pi /2)=-P_{y}(\varphi =3\pi /2)\neq 0$. The direction of the transverse flow will alternate while moving along the *y*-axis. For instance, if at *m* = 1 the energy flow on the *y*-axis is directed towards the center, then at *m* = 2, it will be directed from the center. Summing up, at *m* = 1, the transverse energy flow will be directed from the center on the *x*-axis and towards the center on the *y*-axis. This can occur if the transverse energy flow rotates anticlockwise in quadrants I and III, rotating clockwise in quadrants II and IV. Next, at *m* = 2 on the vertical axis, the transverse energy flow will change sign, becoming directed from the center, while remaining being directed from the center on the horizontal *x*-axis. This can occur if in the four quadrants there are four lines (at an angle of 45°) along which the energy flow is directed to the center. Thus, at *m* = 2, eight transverse energy vortices will be generated (two in each quadrant), featuring alternating (clockwise and anticlockwise) handedness. Using a similar reasoning, it can further be shown that at an arbitrary *m*, there will be 4*m* vortices of energy flow at the focus. The vortex handedness will change to the opposite in passing from one vortex to the other. 

For simplicity, below, we analyze particular cases of Equation (11). From Equation (11), it also follows that at *m* = 0 (linear polarization), the transverse energy flow components equal zero at the focus: *P_x_* = *P_y_* = 0. This can be checked by directly substituting *m* = 0 into Equation (11) and considering the properties of the integrals in Equation (4): *I_p_*,*_−q_*=(−1)*^q^I_p,q_*. At *m* > 0, there is a non-zero transverse energy flow of Equation (11). Let us recall that for the *m*th-order cylindrical polarization, the transverse energy flow at the focus is always zero [33]. At *m* = 1 (circular azimuthal polarization), we can derive from Equation (11) the following expressions for the projections of the energy flow:

(13) $$\begin{array}{l} P_{x}=\frac{1}{4}[I_{0,1}\left(I_{1,2}+I_{1,0}\right)\cos\varphi +I_{1,2}I_{2,3}\sin2\varphi \sin3\varphi +\\ \;\;\;\;\;\;\;\;\;\;+I_{1,0}I_{2,3}\cos3\varphi -I_{1,2}I_{2,1}\cos2\varphi \cos\varphi ],\\ P_{y}=\frac{1}{4}[I_{0,1}\left(I_{1,2}\sin3\varphi -I_{1,0}\sin\varphi \right)+\\ \;\;\;\;\;\;\;\;\;+I_{1,2}I_{2,3}\cos2\varphi \cos3\varphi -I_{1,0}I_{2,3}\sin3\varphi +\\ \;\;\;\;\;\;\;\;\;+I_{1,2}I_{2,1}\cos2\varphi \sin\varphi ],\\ P_{z}=\frac{1}{4}\left(I_{0,1}^{2}-\frac{1}{2}I_{2,3}^{2}-\frac{1}{2}I_{2,1}^{2}\right). \end{array}$$

From Equation (13), the longitudinal energy flow component is seen to be ring-shaped, with the on-axis intensity null. The transverse energy flow components are non-zero and devoid of radial symmetry. From Equation (13), the transverse components of the Poynting vector at the focus are seen to have the following structure:

(14) $$\begin{array}{l} \varphi =0:\;\;\;\;\;P_{x}=A+B>0,\;P_{y}=0,\\ \varphi =\pi /2:P_{x}=0,P_{y}=-A+B<0,\\ \varphi =\pi :\;\;\;\;\;P_{x}=-(A+B)<0,\;P_{y}=0,\\ \varphi =3\pi /2:P_{x}=0,P_{y}=A-B>0,\\ A=I_{0,1}\left(I_{1,2}+I_{1,0}\right)/4,\;B=I_{1,0}I_{2,3}-I_{1,2}I_{2,1}. \end{array}$$

From Equation (14), the energy flow in the focal plane on the horizontal *x*-axis is seen to be directed along the *x*-axis from the center, being directed towards the center on the vertical *y*-axis. This effect occurs if the transverse energy flow rotates anticlockwise in quadrants I and III, rotating clockwise in quadrants II and IV.

One more general conclusion can be made from Equation (11) without the need to carry out numerical simulation. In the relationship for the projection of the Poynting vector in Equation (11), the sine function $P_{y}$ with the maximal spatial frequency is given by sin(2m+1)φ. Hence, at a given *r*, the integrals from Equation (4) that enter Equation (11) will take constant values, with the entire expression for $P_{y}$ being dependent only on the angle *φ*, such that after one full circle of radius *r* in the focal plane, the value of $P_{y}$ will change sign 2(2*m* + 1) times.

### 2.3. SAM in the Strong Focus of a Field with Hybrid Polarization 

First, we recall that the longitudinal projection of the spin density vector or SAM vector equals zero for any *m*th-order cylindrical polarization of the initial field [33]. In this section, we will demonstrate that given the hybrid polarization of Equation (1), the longitudinal projection of the SAM vector in the focus will be non-zero. Hence, let us define the SAM vector in the form [35]:

(15) S=Im(E*×E)

where Im is the imaginary part of the number. Substituting the E-field projections from Equation (2) into Equation (15) yields the longitudinal component of SAM:

(16) $$\begin{array}{l} S_{z}=\frac{1}{4}[I_{0,m}\left(I_{2,m+2}-I_{2,m-2}\right)\sin2\varphi +\\ \;\;\;\;\;\;\;\;\;\;\left(I_{0,m}^{2}-I_{2,m-2}I_{2,m+2}\right)\sin\left(2m\varphi \right)]. \end{array}$$

Equation (16) suggests that at *m* = 0 (linear polarization), $S_{z}=0.$ At *m* = 1, Equation (16) is rearranged to:

(17) $$S_{z}=\frac{1}{4}[I_{0,1}\left(I_{2,3}+I_{2,1}\right)+\left(I_{0,1}^{2}+I_{2,1}I_{2,3}\right)]\sin2\varphi .$$

From Equation (16), the on-axis projection of the SAM vector in the focal plane changes its sign 4*m* times, because Equation (16) contains the function sin(2*mφ*). Hence, there will be 4*m* local vortices of the transverse flow and 4*m* local regions with the positive or negative longitudinal projections of the SAM vector. Notably, in the focal plane regions of anticlockwise handedness of the transverse energy flow, the polarization vector also rotates anticlockwise, meaning that the projection of the SAM vector is positive ($S_{z}>0$), and vice versa—in the focal plane’s local regions of the clockwise handedness of the transverse energy flow, the polarization vector also rotates clockwise, meaning that the longitudinal projection of the SAM vector is negative ($S_{z}<0$). If placed in the focal plane, dielectric microparticles that are slightly lesser in size than the local region under analysis will start rotating around their axis. It is interesting that particles in the adjacent regions will rotate in the opposite directions.

## 3. Results and Discussion

### 3.1. Results of the Numerical Simulation of Focusing Light with Hybrid Polarization 

**Figure 1:**
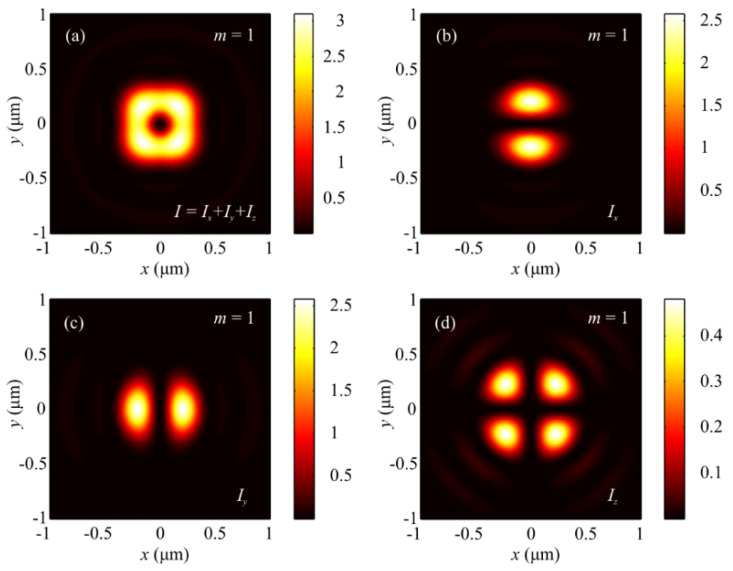
Patterns of (**a**) intensity and its components (**b**) *I_x_*, (**c**) *I_y_*, and (**d**) *I_z_* in the focal plane when focusing a plane wave with the hybrid polarization of Equation (1) at *m* = 1.

**Figure 2:**
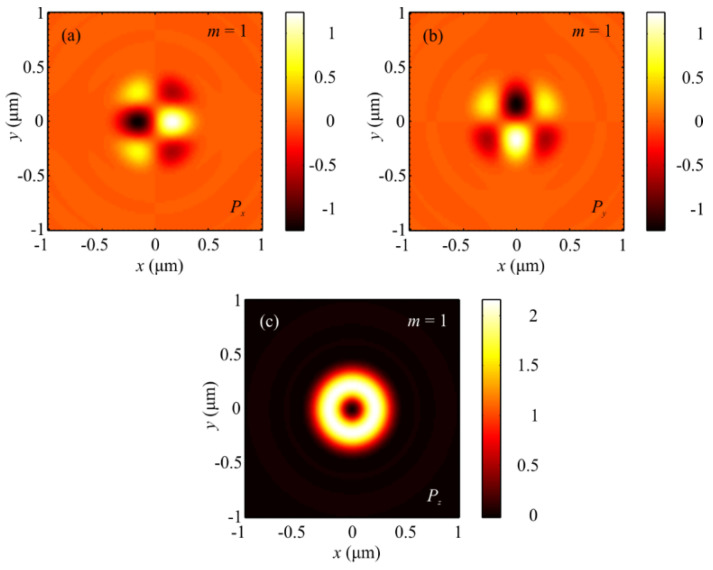
Patterns of the Poynting vector components (**a**) *P_x_*, (**b**) *P_y_*, and (**c**) *P_z_* in the focal plane when focusing a plane wave with the hybrid polarization of Equation (1) at *m* = 1.

The numerical simulation based on the Richards–Wolf formulae [1] was conducted for focusing a 532 nm plane wave with hybrid polarization (Equation (1)) by means of an aplanatic objective lens with NA = 0.95. Figure 1 and Figure 2 depict intensity patterns and the Poynting vector components *P_x_*, *P_y_*, and *P_z_* in the focal plane when focusing a plane wave with the hybrid polarization of Equation (1) at *m* = 1. From Figure 1, the intensity is seen to have 2(*m* + 1) = 2(1 + 1) = 4 local maxima located at the corners of a square-shaped contour. At the focal spot center, there occurs an intensity null. Shown in Figure 2a,b are distributions of the transverse energy flow and the transverse projections (a) *P_x_*, (b) *P_y_* of the Poynting vector. From Figure 2a,b, the energy flow is seen to change its sign 2(2*m* + 1) = 6 times per one full circle around the center. Figure 2c shows the longitudinal projection *P_z_* of the Poynting vector, which is ring-shaped and has a zero value at the center. The patterns in Figure 1 and Figure 2 confirm the conclusions made on the basis of the theoretically derived relationships for the intensity in Equation (9) and the energy flow in Equation (11).

**Figure 3:**
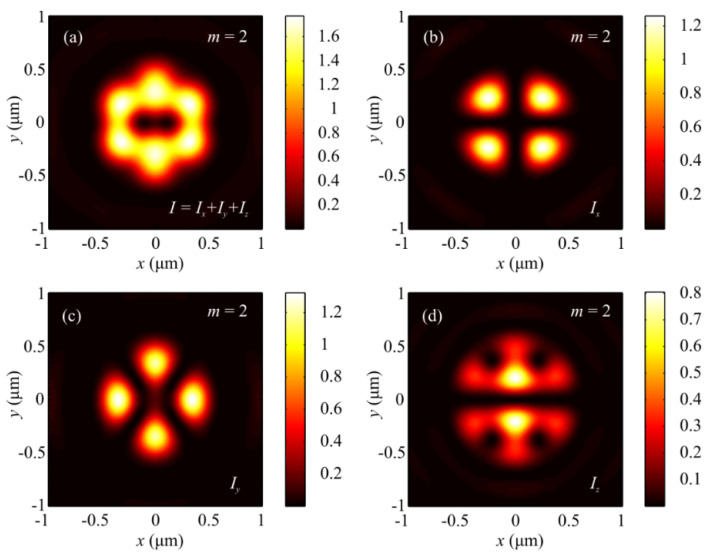
Patterns of (**a**) intensity and its components (**b**) *I_x_*, (**c**) *I_y_*, and (**d**) *I_z_* in the focal plane when focusing a plane wave with the hybrid polarization of Equation (1) at *m* = 2.

**Figure 4:**
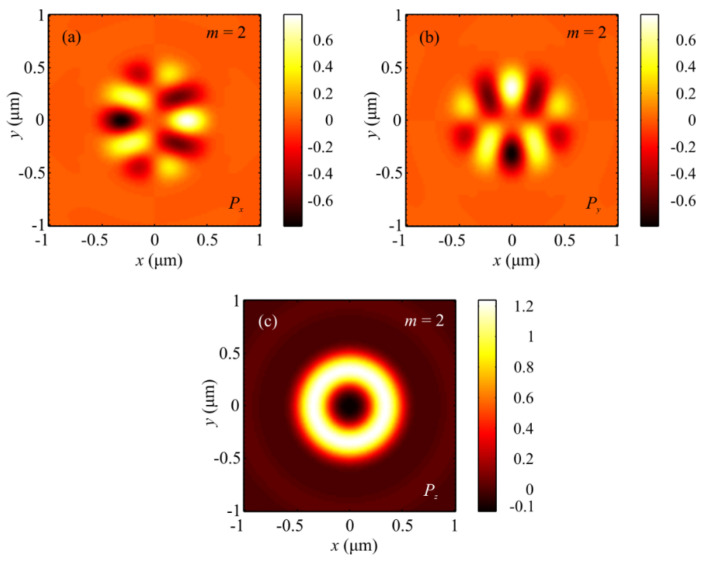
Patterns of the Poynting vector components (**a**) *P_x_*, (**b**) *P_y_*, and (**c**) *P_z_* in the focal plane when focusing a plane wave with the hybrid polarization of Equation (1) at *m* = 2.

Figure 3 and Figure 4 depict patterns of intensity and the projections *P_x_*, *P_y_*, and *P_z_* of the Poynting vector in the focal plane when focusing a plane wave with the hybrid polarization of Equation (1) at *m* = 2. The numerically simulated patterns in Figure 3 and Figure 4 corroborate theoretical predictions that follow from Equations (9) and (11). In fact, the intensity distribution in Figure 3a is seen to have 2(*m* + 1) = 6 local maxima lying on a closed curve around the center. Figure 4a,b depict distributions of the transverse projections *P_x_* (a) and *P_y_* (b) of the Poynting vector, from which the energy flow is seen to change its sign 2(2*m* + 1) = 10 times per full circle around the center. Figure 4c depicts the longitudinal projection *P_z_* of the Poynting vector in the form of a ring. The central energy flow is negative and equal to $P_{z}(r=z=0)=-I_{2,0}^{2}/4$, as seen from Equation (11).

**Figure 5:**
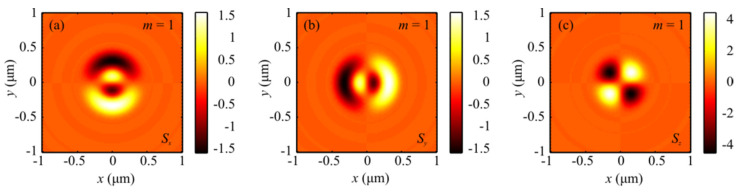
Distribution of SAM vector components *S_x_* (**a**), *S_y_* (**b**), and *S_z_* (**c**) when focusing a plane wave with the hybrid polarization of Equation (1) at *m* = 1.

Figure 5 depicts distributions of the SAM components (a, b, c) *S_x_*, *S_y_*, and *S_z_* when focusing a plane wave with the hybrid polarization of Equation (1) at *m* = 1. From Figure 5c, the longitudinal component of the SAM vector changes its sign 4*m* = 4 times, as seen from Equation (17).

**Figure 6:**
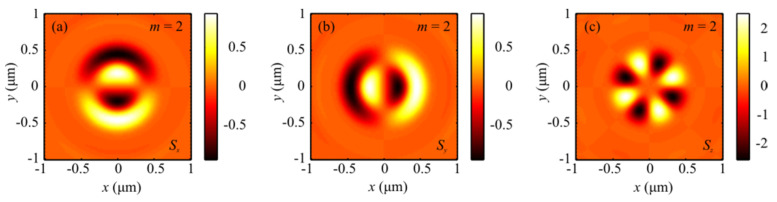
Distribution of the SAM components (**a**–**c**) *S_x_*, *S_y_*, and *S_z_* when focusing a plane wave with the hybrid polarization in Equation (1) at *m* = 2.

Figure 6 shows distributions of the SAM vector components (a, b, c) *S_x_*, *S_y_*, and *S_z_* when focusing a plane wave with the hybrid polarization of Equation (1) at *m* = 2. From Figure 6c, the longitudinal projection of the SAM vector can be seen to be equal to 4*m* = 8, which follows from Equation (17).

**Figure 7:**
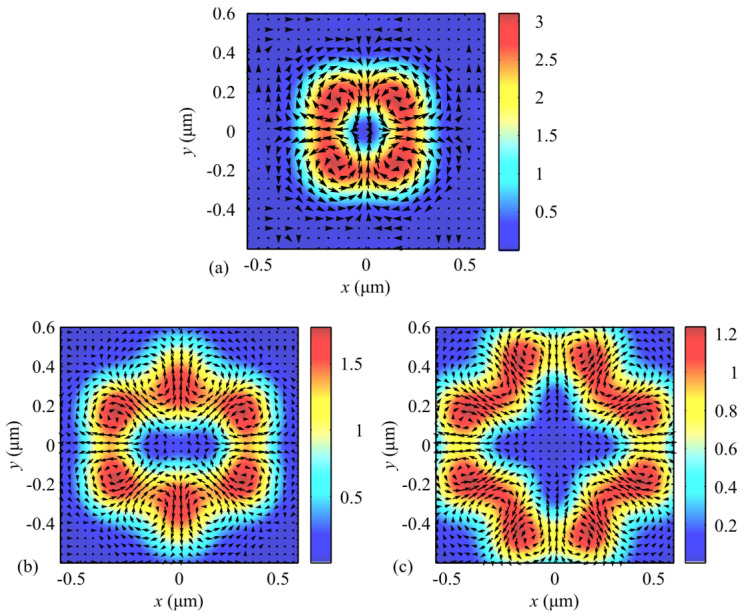
Intensity distribution and the magnitude and direction of the Poynting vector (arrows) in the focal plane when focusing a plane wave with the hybrid polarization of Equation (1) for (**a**) *m* = 1, (**b**) *m* = 2, and (**c**) *m* = 3.

Figure 7 depicts intensity distributions when focusing a plane wave with hybrid polarization in Equation (1) at (a) *m* = 1, (b) *m* = 2, and (c) *m* = 3, with arrows marking the direction of the transverse Poynting vector component in the focal plane. From Figure 7, the number of the transverse flow vortices equals (a) 4*m* = 4, (b) 4*m* = 8, and (c) 4*m* = 12, as can be inferred from Equation (11) for the transverse Poynting vector components. From Figure 7, the centers of the transverse energy flows at the focus can also be seen to be non-coincident with the local intensity maxima. The vortices are centered at points where the transverse energy flow is zero. From the comparison of Figure 6c and Figure 7b, the number of regions with the positive- and negative-valued longitudinal SAM projections (4*m* = 8) is the same as that of the transverse energy vortices (4*m* = 8). A comparison of Figure 6c and Figure 7b also suggests that the longitudinal SAM component is positive (*S_z_* > 0) in the regions of anticlockwise handedness of the transverse energy flow, and vice versa—the longitudinal SAM component is negative (*S_z_* < 0) in the regions of clockwise handedness of the transverse energy vortex. Thus, the polarization vector in the focal plane rotates anticlockwise in the regions where the transverse energy flow also has anticlockwise handedness, and vice versa—the polarization vector rotates clockwise where the transverse energy flow has clockwise handedness. This is in good agreement with the spin-orbital conversion effect. This spatial separation at the focus of left and right circular polarization is a manifestation of the optical spin Hall effect [36]. 

### 3.2. Experiment

**Figure 8:**
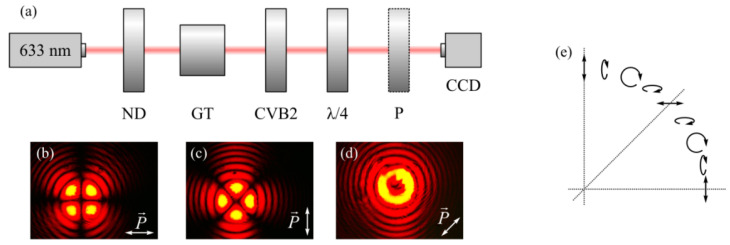
(**a**) Optical setup for the generation and registration of a hybrid polarized beam (Equation (1)) of the second-order *m* = 2, with the analyzer placed (**b**) horizontally (*θ* = 0 degrees), (**c**) vertically (*θ* = 90 degrees), and (**d**) at an angle of *θ* = 45 degrees. (**e**) The experimentally reconstructed dependence of the polarization state on the polar angle *φ* in quadrant I.

Figure 8a shows an optical setup for generating the beam (Equation (1)) with *m* = 2. Figure 8b–d show images of the resulting beam. Light from a Cobolt 06-MLD laser (*λ* = 633 nm, 200 mW) propagates through a neutral-density filter (ND) and a Glan–Taylor polarizer (GT). The resulting linearly polarized light propagates through a vortex half-wave plate (Thorlabs, WPV10-633), which transforms linearly polarized light into a second-order cylindrical vector beam. Finally, the beam propagates through a quarter-wave plate. The resulting beam was registered by a CCD camera (UCMOS 10000KPA).

ND is a neutral density filter, GT is a Glan–Taylor polarizer, CVB2 is a vortex half-wave plate (Thorlabs, WPV10-633), *λ*/4 is a quarter-wave plate, and CCD is a UCMOS 10000KPA camera). In the images of the beam (b)–(d), a linear polarizer–analyzer P was placed before the CCD camera, and was rotated by an angle *θ* equal to 0° (b), 90° (c), or 45° (d).

To be sure that the experimentally obtained beam has the desired hybrid polarization, we simulated an insertion of a linear polarizer into the beam using the Jones calculus formalism. After passing through the linear polarizer–analyzer, the polarization of the beam changes as follows:

(18) $$\left(\begin{array}{c} E_{x,out}\\ E_{y,out} \end{array}\right)=\left(\begin{array}{cc} \cos^{2}\theta & -\sin\theta \cos\theta \\ -\sin\theta \cos\theta & \sin^{2}\theta \end{array}\right)\left(\begin{array}{c} E_{x,in}\\ E_{y,in} \end{array}\right)$$

where *E_x__,in_* and *E_y__,in_* are the electric field components before the polarizer (calculated by the Richards–Wolf formulae), *E_x__,out_* and *E_y__,out_* are the electric field components after the polarizer–analyzer, and *θ* is the angle between the *x*-axis and the polarizer axis.

**Figure 9:**
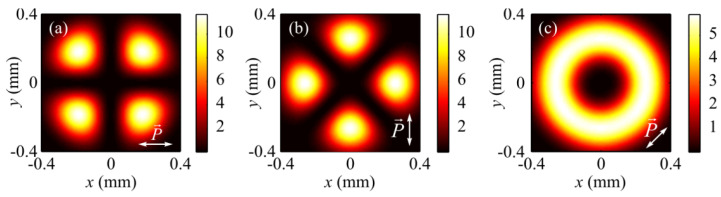
Intensity distribution (simulation) of a hybrid beam (Equation (1)) with *m* = 2 propagated through a linear polarizer–analyzer rotated by the angle *θ* equal to 0 (**a**), *π*/2 (**b**), and *π*/4 (**c**).

Figure 9 shows an intensity distribution of the hybrid polarized beam in Equation (1) at *m* = 2, propagated through a linear polarizer–analyzer, which is rotated by an angle of 0° (Figure 9a), *π*/2 (Figure 9b), or *π*/4 (Figure 9c). From Equation (1), it follows that at the angle $\varphi =\frac{\pi }{4}+\frac{\pi n}{2}$ (along the diagonal lines) there remains only the *E_x_*-component, such that $E=\left(\begin{array}{l} -i\\ 0 \end{array}\right)$; however, at $\varphi =\frac{\pi n}{2}$ (along the Cartesian axes), there remains only the *E_y_*-component: $E=\left(\begin{array}{l} 0\\ 1 \end{array}\right)$.

From a comparison of Figure 8 and Figure 9, the numerically simulated and experimentally generated beams can be seen to have the same polarization. We can also see that in the experiment presented in Figure 8b,c, the intensity distribution is consistent with the calculated intensity distribution of the transverse components of the electric field at the sharp focus of the hybrid beam (1) at *m* = 2, as shown in Figure 3b,c, respectively. Thus, the transverse intensity distribution of the hybrid field (Figure 9a,b) retains its shape at the sharp focus (Figure 3b,c).

**Figure 10:**
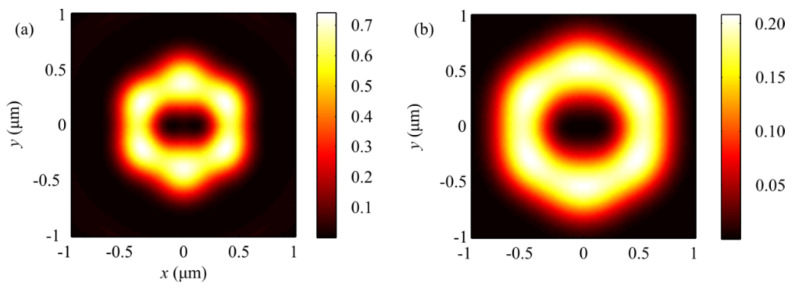
Changes in the focal spot intensity for the light field (Equation (1)) at *m* = 2, with decreasing numerical aperture of the lens to (**a**) *NA* = 0.8 and (**b**) *NA* = 0.6. The focal spot generated with a lens with *NA* = 0.95 is shown in Figure 3a.

Figure 10 shows the calculated intensity distributions at the focus of an ideal spherical lens for the initial field (Equation (1)) at *m* = 2 for different numerical apertures: 0.8 (a) and 0.6 (b). It can be seen from Figure 10 that, as the numerical aperture decreases, the contrast of the local maxima decreases, and the focal spot becomes more and more like a ring.

### 3.3. Optical Setup and Metalens

**Figure 11:**
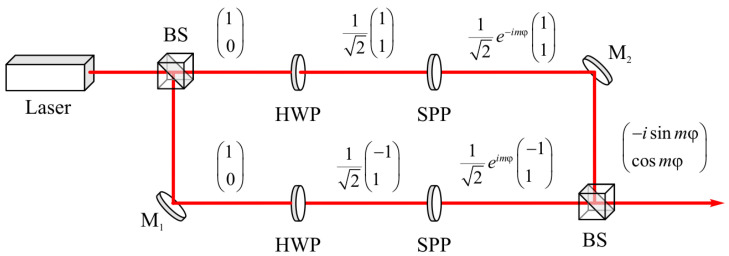
An optical scheme for generating a laser beam with hybrid polarization (Equation (1)) of an arbitrary order *m*. HWP is a half-wave plate; BS is a beam splitter; M1 and M2 are mirrors; SPP is a spiral phase plate.

In the general case, a laser beam with hybrid polarization (Equation (1)) of an arbitrary order *m* can be formed using the optical scheme shown in Figure 11.

A linearly polarized light from a laser (Figure 11) is divided by a beam splitter (BS) into two identical beams that propagate in different arms of a Mach–Zehnder interferometer. Two beams with mutually orthogonal polarization are generated after passing through half-wave plates (HWPs) tilted at different angles. Both of these beams pass through the spiral phase plates (SPPs) with transmission functions exp(*imφ*) and exp(−*imφ*). These beams are combined into one beam with hybrid polarization (Equation (1)) at the interferometer output.

The justification for the choice of the optical scheme in Figure 11 follows from the chain in the Jones vector (Equation (1)) transformations:

(19) $$E=\left(\begin{array}{l} -i\sin m\varphi \\ \cos m\varphi \end{array}\right)=\left(\begin{array}{l} -i\frac{e^{im\varphi }-e^{-im\varphi }}{2i}\\ \;\;\frac{e^{im\varphi }+e^{-im\varphi }}{2} \end{array}\right)=\frac{1}{2}\left(\begin{array}{l} -e^{im\varphi }+e^{-im\varphi }\\ \;\;e^{im\varphi }+e^{-im\varphi } \end{array}\right)=\frac{1}{2}e^{im\varphi }\left(\begin{array}{l} -1\\ \;\;1 \end{array}\right)+\frac{1}{2}e^{-im\varphi }\left(\begin{array}{l} 1\\ 1 \end{array}\right).$$

The experiments in Figure 8 at *m* = 2, along with the optical scheme in Figure 11, make it possible to form laser beams with hybrid polarization (Equation (1)) in practice. However, they are not related to the sharp focusing of such beams. The sharp focusing of such fields can be realized in practice using a micro-objective with a high numerical aperture (for example, a 100 × objective lens (100X Mitutoyo Plan Apo Infinity Corrected Long WD Objective)). The intensity distribution and the energy flux at the focus can be measured with subwavelength resolution using a near-field scanning optical microscope with a resolution of ~35 nm (for example, SNOM_C, NT-MDT). The authors plan to conduct such an experiment in the near future. 

**Figure 12:**
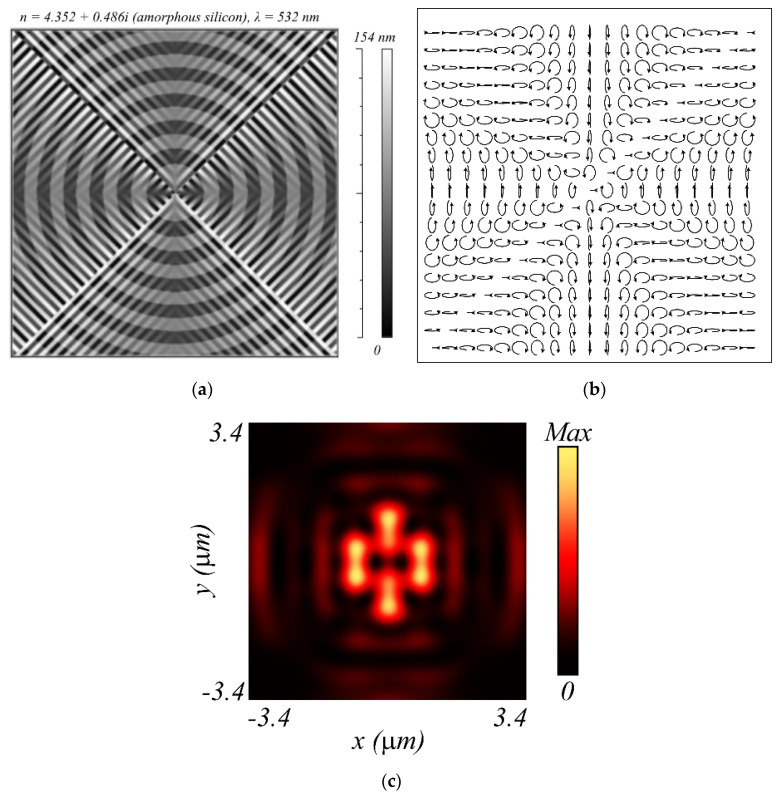
A subwavelength relief of the metalens surface (black indicates depressions, white indicates elevations) (**a**) in a thin film of amorphous silicon (film thickness 154 nm), which forms a light field with hybrid polarization (Equation (1)) (**b**) and the intensity distribution (**c**) in the focal plane at a 500 nm distance behind the metalens upon illumination by the linearly polarized light. The metalens has an *NA* of ~1.

Additionally, we calculated the subwavelength profile of the metalens in Figure 12a, which can generate a field with hybrid polarization (Equation (1)) of order *m* = 2 immediately behind this lens (Figure 12b) when illuminated by a Gaussian beam with linear polarization. The focus is generated behind the metalens at a wavelength distance of 532 nm. Figure 12c shows the intensity distribution in the focal plane calculated by a finite-difference time-domain method (FDTD method). It can be seen that the intensity distribution (Figure 12c) is consistent with the intensity calculated according to the Richards–Wolf theory (Figure 3a). The metalens size (Figure 12a) is 6.7 × 6.7 μm. This size is convenient for modeling, but can be increased to any other size. It is assumed that the metalens is made from an amorphous silicon film with a refractive index of 4.352 + *i*0.486 for a wavelength of 532 nm; it combines the functionalities of three different optical elements: a polarizer, a phase corrector, and a microlens (Fresnel zone plate). The polarizer rotates the incident linear polarization vector by a preset angle and performs the linear-to-elliptical polarization conversion. The polarizer is realized by subwavelength diffraction gratings with different line slopes and different heights; the period of all local gratings is 184 nm. The polarizer consists of eight sectors, in each of which the slope of the lines is constant. A phase corrector is a substrate with a smoothly varying relief height to provide the required phase at various points of the metalens. The Fresnel zone plate provides sharp focusing of laser light at a near-wavelength distance. All three of these components form the final profile of the metalens heights; its maximum height difference is 154 nm. The polarizer and the phase corrector under illumination by a plane wave with linear polarization *E_y_* create a polarization distribution at the output, as shown in Figure 12b, and the entire metalens converts the *E_y_* wave into a focal spot at a 500 nm distance, with its intensity distribution depicted in Figure 12c. It can be seen that the intensity distribution at the focus of the metalens (Figure 12c) is in good agreement with the calculated intensities in Figure 3a and Figure 7b. Both focal spots (in Figure 3a and Figure 12c) have 2(*m* + 1) = 6 local maxima.

The metalens transmission function (Figure 12a) without the Fresnel zone plate is described by the Jones matrix, which is the product of the rotation matrix of the linear polarization vector, the angle 2*φ*, and the matrix of a quarter-wave plate, which converts the linear polarization to elliptical or circular polarization.

(20) $$R\left(\phi \right)=\left(\begin{array}{cc} i\cos2\phi & -i\sin2\phi \\ \sin2\phi & \cos2\phi \end{array}\right)=e^{i\pi /4}\left(\begin{array}{cc} e^{i\pi /4} & 0\\ 0 & e^{-i\pi /4} \end{array}\right)\left(\begin{array}{cc} \cos2\phi & -\sin2\phi \\ \sin2\phi & \cos2\phi \end{array}\right).$$

The calculated ultrathin metalens (Figure 12a) with a high numerical aperture and a small diameter, which alone replaces the entire optical scheme in Figure 8a (and also replaces a focusing lens with a numerical aperture close to 1), may serve as a prototype of a compact focusing sensor for optical topography measurement of rough surfaces [37].

## 4. Conclusions

In this work, the tight focusing of laser beams with *m*th-order circular–azimuthal polarization was analyzed. This is a new type of inhomogeneous polarization which combines properties of the *m*th-order cylindrical and circular polarizations. Using the Richards–Wolf formalism, we deduced analytical expressions to describe the projections of the E- and H-vectors, the intensity distribution, and the projections of the Poynting vector and SAM vector in the tight focus of light. We showed theoretically and numerically that the intensity pattern in the focal spot has 2(*m* + 1) local maxima located along a closed contour centered at the on-axis zero-intensity point. We also showed that at the focus there are 4*m* vortices of the transverse energy flows with their centers located between the local intensity maxima, while the transverse energy flow vortex changes its handedness 2(2*m* + 1) times per full circle around the optical axis. Interestingly, the longitudinal SAM component at the focus changes its sign 4*m* times. The longitudinal SAM component was shown to be positive in the regions of anticlockwise handedness of the transverse energy vortex, with the polarization vector rotating anticlockwise around the optical axis, and vice versa—the polarization vector rotates clockwise and the longitudinal SAM component is negative in the regions where the energy flow rotates clockwise. The possibility of generating a second-order hybrid beam with a vortex half-wave plate was experimentally demonstrated. The coincidence between the experimentally generated beam and those studied theoretically was verified using the Jones formalism. The results obtained in terms of controlling the intensity maxima allow for the transverse mode analysis of laser beams in waveguide-based sensors [38,39]. This kind of CVB can be used in phase-sensitive surface plasmon resonance biosensors with high resolution [40], or in graphene biosensors for real-time subcellular imaging [41]. Other applications include Raman spectroscopy [42] and vector magnetic field sensing [43].

## Data Availability

Code underlying the results presented in this paper is available in [44].
